# A Regional Climate Mode Discovered in the North Atlantic: Dakar Niño/Niña

**DOI:** 10.1038/srep18782

**Published:** 2016-01-07

**Authors:** Pascal Oettli, Yushi Morioka, Toshio Yamagata

**Affiliations:** 1 Application Laboratory, Japan Agency for Marine-Earth Science and Technology, Yokohama, 236-0001, Japan

## Abstract

The interrannual variability of coastal sea surface temperature (SST) anomalies confined off Senegal is explored from a new viewpoint of the ocean-land-atmosphere interaction. The phenomenon may be classified into “coastal Niño/Niña” in the North Atlantic as discussed recently in the Northeastern Pacific and Southeastern Indian Oceans. The interannual variability of the regional mixed-layer temperature anomaly that evolves in boreal late fall and peaks in spring is associated with the alongshore wind anomaly, mixed-layer depth anomaly and cross-shore atmospheric pressure gradient anomaly, suggesting the existence of ocean-land-atmosphere coupled processes. The coupled warm (cold) event is named Dakar Niño (Niña). The oceanic aspect of the Dakar Niño (Niña) may be basically explained by anomalous warming (cooling) of the anomalously thin (thick) mixed-layer, which absorbs shortwave surface heat flux. In the case of Dakar Niña, however, enhancement of the entrainment at the bottom of the mixed-layer is not negligible.

Equatorial coupled ocean-atmosphere phenomena exist in all tropical oceans: El Niño Southern Oscillation (ENSO), with variations, in the Pacific, the Indian Ocean Dipole (IOD) in the Indian Ocean and the Atlantic Niño in the Atlantic. The prominent phenomena as well as their impacts have been highly documented in past decades. However, regional SST anomalies associated with anomalous atmospheric conditions in nearshore regions have not received much attention except for the Benguela Niño^1^,^2^ despite their importance in regional climate. Recently, the existence of the abnormal SST warming off Baja California, together with its dependence/independence with ENSO, has recently been discussed in detail by Yuan and Yamagata^3^ and called California Niño^3^ as coastal Niño due to regional positive feedback processes among ocean, land and atmosphere. In Fig. 1, we also notice another anomalously warm coastal SST region off Senegal in the North Atlantic (10° to 26°N). This phenomenon was noticed already for the period 1957–1995 by Roy and Reason^4^ in 2001. Since the Pacific-Atlantic connection through anomalous Walker circulation appears to develop during the early part of the year^5^, such an atmospheric bridge between Pacific and Atlantic basins may explain such a delayed influence of ENSO to some extent. However, considering the recent work on the California Niño^3^, the variability of coastal SST anomalies confined off Senegal needs to be readdressed with a possible view of a coastal Niño. This is the motivation of the present report.

The North Atlantic subtropical gyre is one of the five major oceanic subtropical gyres. It is bounded by the Gulf Stream in the west, the North Atlantic Current in the north, the Canary Current in the east and the North Equatorial Current in the south. The eastern boundary extends from the north of the Iberian Peninsula at 43°N to the south of Senegal at 10°N^6^. From 30°N to 10°N, the Canary Current continuously flows all year round toward the equator^7^,^8^, which is associated with climatological surface winds blowing southward along the Northwest African coast^9^. Surface and subsurface waters are relatively cool^10^, owing to the upwelling due to Ekman divergence^11^ driven by the trade winds. Along the coast of Senegal, northerly winds transport coastal surface water into the interior ocean^12^. This is counterbalanced by upwelled cold and nutrient-rich water^10^, generating one of the major eastern boundary upwelling systems^6^, with an estimated primary production above 500 mgC m^−2^ d^−1^ in the region^13^.

Along the northwest African coast, this upwelling system represents a rich fishing ground^14^, dominated by pelagic and demersal fish species. From the mid-1970s to mid-2000s, total landings fluctuated between 1.3 Mt and 2.6 Mt^6^. During the same period, the total landings of sardines (*Sardinella maderensis* and *Sardinella aurita*) for Senegal varied between 59 kt and 268 kt^15^. These fluctuations may be seen as the results of the combination of changes in the exploitation techniques as well as in the natural variability of the upwelling intensity^4^,^16^. The latter is related to seasonal and interannual variations of regional winds as well as the large-scale trade winds. The seasonal meridional shift of the trade winds, due to the seasonal migration of the Inter-Tropical Convergence Zone (ITCZ), induces seasonal variations in the upwelling system^6^,^8^,^9^,^12^,^17^,^18^,^19^,^20^ along the eastern boundary.

Longer time scale variability has been detected also in the coastal system. In the northern part of the upwelling system, the North Atlantic Oscillation (NAO) modulates wind conditions and fish catch^21^. In the southern part, and particularly off Senegal, a periodicity of around 16 years exists in the surface wind^12^, compatible with the influence of the Atlantic Meridional Mode (AMM) in this region^22^. The AMM is the dominant source of coupled ocean-atmosphere variability in the Atlantic.

We first focus on the sea surface temperature variability south of the Cap-Vert in Senegal and then study whether a coupled ocean-land-atmosphere phenomenon exists or not. We show for the first time that a regional ocean-land-atmosphere coupled climate phenomenon really exists off the coast of Senegal just like those along similar eastern boundaries of different ocean basins^3^,^23^,^24^. In the present short article, we do not study possible triggers and connections with other climate modes in detail; we expect further studies of this interesting phenomenon.

## Results

**Figure 2:**
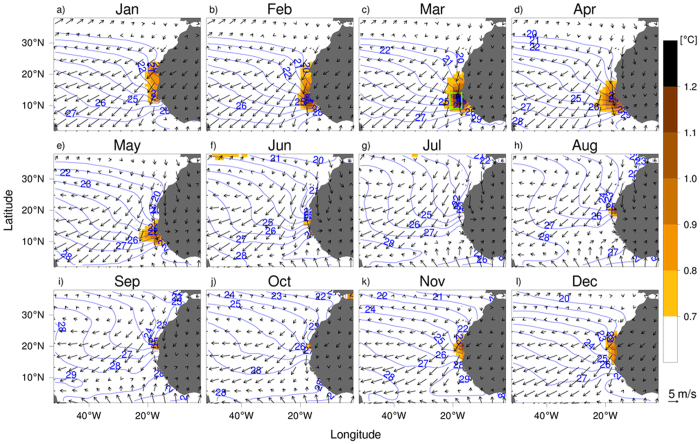
Monthly SST climatology (contour) and its variability (shading), with 10-m winds (vector). Only isotherms between 20 °C and 30 °C are displayed in blue. The green rectangle in (**c**) is the region selected to study the local SST variability. The figure was plotted by R software.

The monthly variability of the sea-surface temperatures (SST) off West Africa for the period 1982–2011 is shown in Fig. 2; the monthly climatology of surface winds for the same period is shown as well. Between 40°N and 15°N, the climatological winds blow southward all year round along the northwest African coast. The region south of 15°N is influenced by the seasonal migration of the ITCZ, with climatological wind blowing northward from June to September (Fig. 2f–i). The sea surface isotherms are mainly zonal in the central North Atlantic off West Africa. However, those become almost parallel to the coastline of West Africa from June to September, due to the coastal upwelling of cold water^25^. This particular feature may be due to a combination of remote forcing associated with the latitudinal migration of the ITCZ, internal variability related to seasonality of the southward Canary Current, and the Dakar (Guinea) Dome^26^,^27^,^28^. A seasonal displacement of climatological surface isotherms is also seen off the northwest African coast with a marked alongshore gradient of SST. The maximum of regional variability in SST is clearly associated with this alongshore SST gradient. The maximum of SST variability starts in November (Fig. 2k) around 15°N off Senegal. This regional maximum migrates southward and peaks in March (Fig. 2c), and almost vanishes by June (Fig. 2f). March to June marks the upwelling season off Senegal. This confirms the effect of the seasonal ITCZ migration on the coastal SST variability. The present study focuses on this boreal spring maximum, which is captured by a green rectangle defined by 21°–17°W, 9°–14°N in Fig. 2c.

**Figure 3:**
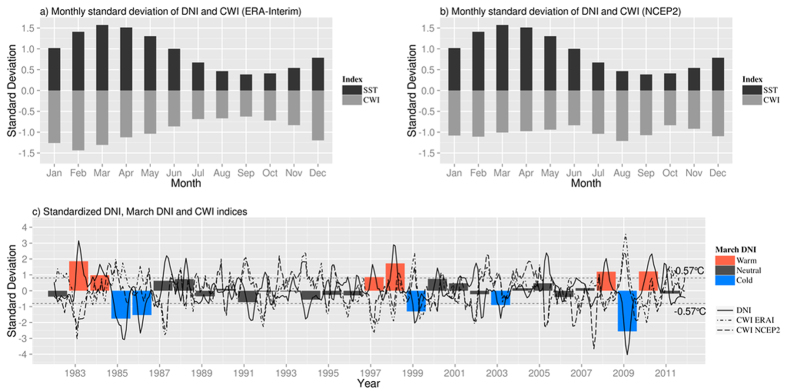
(**a**) Monthly standard deviation of standardized coastal SST index and coastal wind index based on ERA Interim in the region [21°–17°W, 9°–14°N] for the period 1982–2011, (**b**) Same as (a) but with coastal wind index based on NCEP/DOE AMIP II, (**c**) Time series of the standardized coastal SST index (black line), standardized coastal wind index (black two-dash line) and standardized coastal SST index in March (colored bars) in the region [21°–17°W, 9°–14°N] for the period 1982–2011. Colors correspond to identified warm (red), neutral (dark grey) and cold (blue) events. Horizontal dotted lines denote the ± 0.8σ (0.57 °C). The figure was plotted by R software.

We define the Dakar Niño/Niña Index (DNI) as the area-average of SST anomalies in the rectangle (Fig. 3). To characterize the effect of the wind on coastal SST, a coastal wind index (CWI) is constructed by averaging the meridional wind over the same region. The sign of this index is positive (negative) for averaged southward (northward) winds. When it is positive (negative), the trade winds are increased (decreased) locally compared to the climatology of the region. The standard deviation of the normalized DNI and CWI is shown in Fig. 3a,b. In regard to CWI, we have adopted two different reanalysis datasets, ERA Interim and NCEP/DOE AMIP II. We find that the oceanic variable DNI is locked to seasons; the peak variability occurs in March. The atmospheric variable CWI is locked to seasons, as well. The index based on ERA Interim shows the peak variability in February, whereas the one based on NCEP/DOE AMIP II shows two peaks in February and August. The peak in August is not associated with the SST variability at all; it might be related to the seasonal migration of ITCZ. While interesting, interpretation of this peak in August is beyond the scope of the current paper. Interestingly, this peak does not appear in ERA Interim. Hereafter we focus on the CWI peak in February, which is associated with the oceanic variability. This unique feature means that the oceanic and atmospheric variations grow and decline together in the region of interest, and suggests the existence of regional ocean-atmosphere interaction.

As seen in Fig. 3c, the DNI is associated with strong interannual variability. We find even the existence of decadal variability, particularly with a period of less interannual variability between 1987 and 1997. In recognition of the interannual variability, we define Niño- and Niña-like events when the February-March-April (FMA) average of normalized DNI is above (below) 0.8 (−0.8) standard deviation (i.e. ±0.57 °C), respectively. In this way, six warm events (1983, 1984, 1997, 1998, 2008 and 2010) and five cold events (1985, 1986, 1999, 2003 and 2009) are identified for the period 1982–2011. Other years are considered to be neutral. Also, an index of the mixed-layer depth (MLDI) is calculated over the same region. This index represents the variability of the subsurface condition in the coastal upwelling region. In association with the MLDI, a coastal upwelling index (CUI) and an index of Ekman pumping (EKMI) are also prepared. Finally, an index of the short wave flux (SWI) is constructed. To be consistent with those oceanic indices, we adopt CWI based on NCEP/DOE AMIP II hereafter.

**Figure 4:**
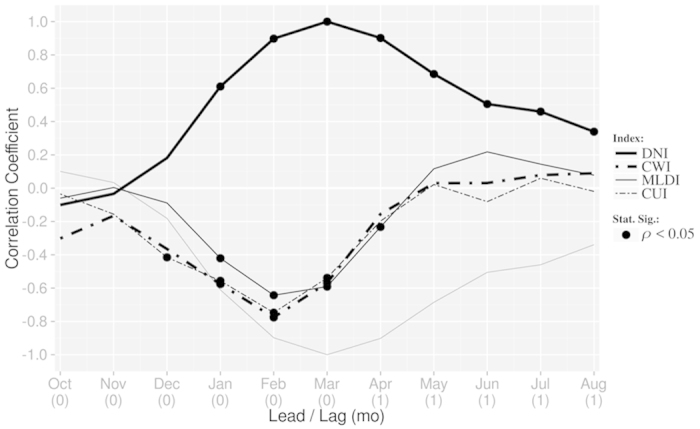
Lead/lag monthly correlation coefficients between DNI and itself, coastal wind (CWI), mixed layer depth (MLDI) and coastal upwelling (CUI) indices, over the 1982–2011 period. The inverted autocorrelation of DNI is also displayed in the figure (grey solid line) for convenience. Correlation coefficients significant at the 95% confidence level (according to a random-phase test) are denoted by a filled circle. The figure was plotted by R software.

We then calculated the autocorrelation of DNI to analyse persistence of the SST signal (Fig. 4, bold solid line) over the period 1982–2011. To capture ocean-atmosphere coupled processes, we also calculated the lead/lag correlation between DNI and other indices described above, i.e. CWI, MLDI and CUI (Fig. 4, bold dot-dash, solid, dashed and two-dash lines, respectively) for the same period. In the calculation, the window is centered on March.

First, we find that the coastal wind variability (Fig. 4, bold dot-dash line) precedes the coastal SST variability by one month; the maximum correlation (−0.79, significant at 95% confidence level, black point) appears in February. The correlation between the wind and the SST rises from November through February, and then declines. The correlation between the coastal SST and the mixed-layer depth (Fig. 4, solid line) shows an interesting behaviour; the latter takes the opposite sign of the former, suggesting the mixed-layer depth variation plays a very important role in determining the SST variation. From November through February, the correlation gradually increases from 0 to −0.64 (significant at 95% confidence level). Then, the coefficient decreases between March and May, and becomes insignificant. The variability of the coastal upwelling (Fig. 4, two-dash line) leads the coastal SST variability, and the maximum correlation −0.76 (significant at 95% confidence level) appears in February. The evolution of correlation coefficients for each of coastal wind, mixed-layer depth and coastal upwelling shows quite a similar behaviour, and the peak correlation occurs in February, one month before the peak month of March for coastal SST. During the evolution phase of DNI from November to February, those three indices are close to the reversed coastal SST, indicating their crucial effect on the SST variability of the present interest.

### Dakar Niño

**Figure 5:**
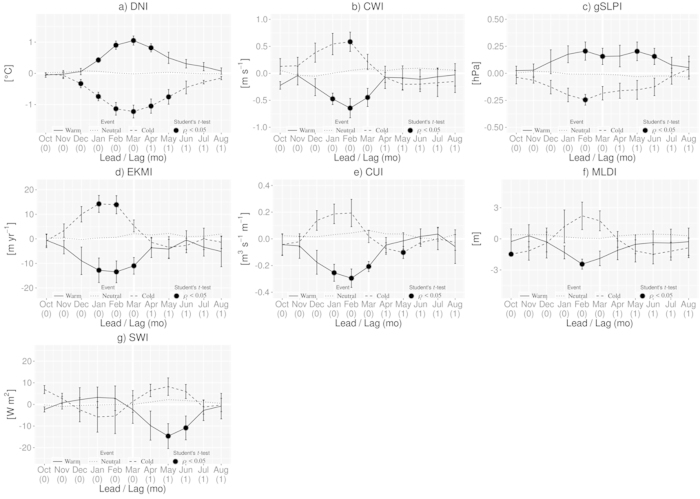
Time series of composite anomalies of the (**a**) DNI SST index, (**b**) CWI (coastal wind index), (**c**) gSLPI (cross-shore pressure gradient index), (**d**) EKMI (Ekman pumping index), (**e**) CUI (coastal upwelling index), (**f**) MLDI (mixed-layer depth) and (**g**) SWI (shortwave radiation) index for warm (solid line) and cold (dashed line) events, and neutral state (dotted line). Solid, dashed and dotted lines are the values of each index during warm events, cold events and neutral state, respectively. Values significant at the 95% confidence level (according to a two-sided Student’s *t*-test with 10,000 permutations) are denoted by a filled circle. The figure was plotted by R software.

Based on the classification of warm, cold and neutral events as shown in Fig. 3b, we have performed composite analysis to extract inherent features. The growing phase of the positive DNI (Fig. 5a, solid line) starts in December and becomes significant in January (0.43 °C). The peak of 1.05 °C is reached in March. The CWI takes a negative peak value of −0.65 m s^−1^ in February, one month earlier than the DNI (Fig. 5b, solid line). The important feature here is that the oceanic variable DNI and the atmospheric variable CWI grow together in the region of interest. This suggests the existence of a regional ocean-atmosphere co-evolution, led initially by the atmosphere.

The climatological mean of pressure over the ocean is larger than that over the land in the region of the present interest. This difference is associated with the climatological southward alongshore winds. To examine roles of cross-shore atmospheric pressure gradient, we have first calculated two area-averaged sea level pressure indices over the ocean [21°–17°W, 9°–14°N] and over the land [15°–11°W, 9°–14°N]. An index of cross-shore pressure gradient (gSLPI) is then derived by subtracting the ocean index from the land index. In the present definition, positive (negative) anomaly of the gSLPI indicates a reversed (enhanced) anomalous cross-shore gradient, which corresponds to northward (southward) alongshore wind. In Fig. 5c (solid line), the anomaly in the gSLPI is positive, which indicates the existence of anomalous northward alongshore winds. In other words, the ocean-land pressure difference is involved in the evolution of the Dakar Niño.

In response to the weaker alongshore wind, compared to the climatology, Ekman pumping anomalies appear (Fig. 5d, solid line) together with inshore Ekman transport anomalies (not shown). This is consistent with the downwelling anomalies (Fig. 5e, solid line). Maximum of Ekman pumping and downwelling occur in January-February. Just in phase with this, the mixed-layer (Fig. 5f, solid line) is anomalously thin, while the anomaly in shortwave radiation (Fig. 5g, solid line) is close to normal condition. This situation favors the effective warming of the surface mixed-layer even with normal shortwave radiation^29^. We have examined the typical Dakar Niño years 1983 and 1998 to illustrate this. These years are the warmest with similar SST anomalies (1.31 °C and 1.22 °C, respectively). In March 1983, the warming of the SST is due to a strong shortwave radiation anomaly (2.37 × 10^7^ °C s^−1^) over a thin mixed-layer (−4.77 m). In March 1998, despite shortwave radiation anomaly close to normal condition (0.11 × 10^7^ °C s^−1^), the thin mixed-layer (−1.71 m) favoured the warming of the SST.

**Figure 6:**
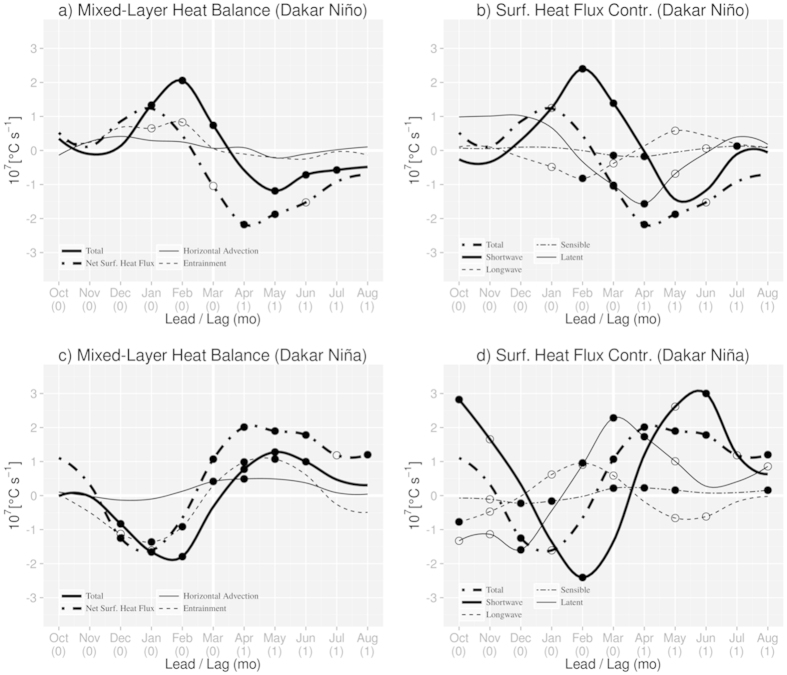
Mixed-layer heat balance during (**a**) Dakar Niño and (**c**) Dakar Niña, and surface heat flux contribution during (**b**) Dakar Niño and (**d**) Dakar Niña. In (**a**,**c**), the time series of composite anomalies of the mixed-layer temperature tendency (bold solid line) and its three component: net surface heat flux (bold two-dash line), horizontal advection (solid line) and entrainment (dashed line). In (**b**,**d**), the time series of composite anomalies of the surface heat flux contribution (bold two-dash line) and its four components: shortwave radiation (bold solid line), longwave radiation (dashed line), sensible heat (two-dash line) and latent heat (solid line) fluxes. Values significant at the 90% and 80% confidence level (according to a two-sided Student’s *t*-test with 10,000 permutations) are denoted by a filled circle and an open circle, respectively. The figure was plotted by R software.

In Fig. 6a, the tendency of the mixed-layer temperature anomaly is diagnosed. During the evolution phase of DNI (around December to March), the tendency (bold solid line) is positive as expected, reaching a peak in February. This is mostly due to the contribution from the net surface heat flux (bold two-dash line), which reaches its maximum in January. Contributions from horizontal advection and entrainment (thin solid line and dashed line, respectively) are rather limited. Decomposing the anomalous net surface heat flux (Fig. 6b), we find that the main contributor is the shortwave radiation (bold solid line). The energy flux between the atmosphere and the ocean (Fig. 6b, solid line) is downward during the evolution phase of DNI, i.e. the atmosphere warms the ocean. We note, however, that this does not necessarily mean increase of the actual shortwave radiation; thinner mixed-layer depth enhances the effective warming role of the shortwave radiation as shown in the previous work^29^. The anomalous latent heat flux is positive and stable between December and January. It becomes null in February, then negative in March (significant at the 90% confident level) during the peak of the DNI, meaning release of energy from the ocean into the atmosphere.

During the decaying phase of the DNI (April to August), the CWI (Fig. 5b, solid line) also decays to zero. The decrease in the northward wind anomaly partially explains the decay of the warm SST anomalies. Associated with the revival of the total wind speed, the mixed-layer becomes less thin (Fig. 5f, solid line). In accord to this, the Ekman pumping and downwelling anomalies weaken to become almost neutral (Fig. 5d,e, solid line). During the same period, the mixed-layer temperature tendency (Fig. 6a, bold solid line) becomes negative. The main contributor is again the anomalous net surface heat flux contribution (Fig. 6a, bold two-dash line), but it is negative in the decaying phase. The decrease in the effective contribution of the shortwave radiation (Fig. 6b, bold solid line) explains that of net surface heat flux. Again, we note that this does not necessarily mean the actual decrease of the shortwave radiation; it is related to the less thin mixed-layer depth. In the decaying phase of DNI, the ocean is losing heat as expected (Fig. 6b, solid line).

### Dakar Niña

From December to March, the negative DNI (Fig. 5a, dashed line) grows from −0.33 to −1.22 °C. In accord to this growth of the SST anomaly, the CWI (Fig. 5b, dashed line) increases just like a mirror image of Dakar Niño. In Fig. 5c (dashed line), the cross-shore pressure gradient anomaly is negative because the SLP over the land is anomalously smaller than that over the ocean. This is consistent with the southward surface wind anomalies. The wind speed anomaly reaches the peak of 0.59 m s^−1^ in February and then start declining in March. The peak of the coastal wind speed is reached one month before that of DNI; this situation is the same with the case for the warm event. Between January and March, the Ekman transport is moving surface waters away from the coast, inducing anomalous Ekman suction (Fig. 5d, dashed line) and amplified upwelling due the the stronger coastal wind (Fig. 5e, dashed line). In February, the upwelling reaches 0.19 m^3^ s^−1^ per meter of coastline. As a response to the CWI enhancement, the mixed-layer becomes thicker through intensified evaporation and mixing (Fig. 5f, dashed line) with a peak anomaly of 2.2 m in February. During the growing phase, anomaly in shortwave radiation (Fig. 5g, dashed line) is negative, but close to the normal condition.

The tendency of the mixed-layer temperature anomaly is diagnosed in Fig. 6c (bold solid line). Between November and February, the tendency is negative. The decrease in the surface heat flux anomalies, with a minimum in February, explains the negative tendency. Just like the warm events, the contribution from anomalous surface net heat flux (bold two-dash line) plays an important role in this. However, the contribution from the anomalous cold water entrainment (dashed line) is also noticeable, particularly between December and February. The contribution of the horizontal advection (solid line) is negligible. Associated with the increase in the wind speed, the ocean also releases heat to the atmosphere due to active evaporation between October and January (Fig. 6d, solid line). The longwave radiation anomaly reaches a peak in February. However, the effective role of the anomalous shortwave radiation (Fig. 6d, bold solid line) is again the main contributor of the anomalous net surface heat flux decrease with a minimum in February (Fig. 6d, bold two-dash line). Again we note this does not necessarily mean actual decrease of the anomalous shortwave radiation. The combination of the above effects leads to decrease of the SST in the study area.

During the decaying phase of the DNI (Fig. 5a, dashed line), the speed of the coastal wind is gradually decreasing and becomes negative (but close to zero) from May to August (Fig. 5b, dashed line). Together with the reduction of the wind speed, the mixed-layer becomes thinner (Fig. 5f, dashed line), while the Ekman suction (Fig. 5d, dashed line) and the coastal upwelling (Fig. 5e, dashed line) is reduced to almost null. The mixed-layer temperature tendency becomes positive after March (Fig. 6c, bold solid line) in accord to the anomalous surface net heat flux (Fig. 6c, bold two-dash line) which is the main contributor to the tendency. After March, the anomalous entrainment (Fig. 6c, dashed line) becomes also positive (significant at the 90% confident level). This may be due to the reduction of the wind speed. During the same period, the anomalous shortwave radiation (Fig. 6d, bold solid line) remains the effective main contributor of the anomalous surface net heat flux. From February, the atmosphere is also warming the upper ocean, as values of the anomalous latent heat flux are positive (Fig. 6d, solid line).

### Remote forcing

**Figure 7:**
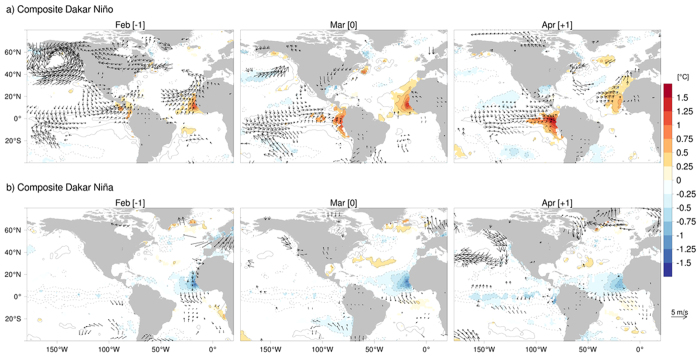
Composite analysis of SST (contour and shading) and 10-m winds (vector) anomalies from February to April, and for the period 1982–2011, during (**a**) Dakar Niño and (**b**) Dakar Niña. Only wind values significant at the 95% confidence level according to a two-sided Hotelling’s T2-test are displayed. Significant SST values at the 95% confidence level (according to a two-sided Student’s *t*-test) are shaded. The figure was plotted by R software.

The interannual variability of SST is confined along the northwest African coast (Fig. 7). The spatial extent shows the maximum in March. In the warm events (Fig. 7a), southwesterly wind anomalies are significant over the study area in February, before the peak in the SST anomalies. Interestingly, El Niño signal is found in the composite in the equatorial Pacific, although it is not significant. Indeed, in the six years identified as coastal warm events, three occurred in the spring season following an El Niño event (1983, 1998 and 2010). This may have influenced the composite picture. Previous studies^4^,^5^ suggested the possible existence of an atmospheric bridge, connecting the north tropical Atlantic to the Equatorial Pacific. However, we must be careful because three other warm events (1984, 1997 and 2008) are not linked with ENSO. The Dakar Niño may be excited through intrinsic regional ocean-land-atmosphere interaction.

The spatial extent of cold SST events is larger than that of the warm SST events, particularly extending westward in the tropical Atlantic (Fig. 7b). We note that the composite pattern of SST anomalies in March and April resembles the typical mid-latitude response to the Atlantic Meridional Mode (AMM)^22^ with poles of negative anomalies around 12°N and 45°N, and a pole of positive anomalies around 30°N. This might suggest the involvement of the AMM on some of the cold events. The year of 2009 corresponds to the strongest Dakar Niña with a negative value (−1.81 °C) in the analysis period. This event seems to coincide with a strong AMM event, which occurred in the same year. The cooling in the equatorial North Atlantic in the first half of 2009 was initiated by stronger than normal trade winds during January and February^30^. But for other Dakar Niña events (1985, 1986 and 1999), the influence of the AMM is not well identified. Dakar Niña events seem to occur even during negative AMM events. In 1994, the state of SST in the region of interest was almost neutral, while a strong AMM event occurred. In regard to Dakar Niño, it appears during positive (2010), negative (1984, 2008) or neutral (1983, 1998) AMM states. Further investigation of the link between the two climate modes is certainly necessary. In Fig. 7b, La Niña signal is also seen in the tropical Pacific; this might suggest involvement of La Niña, as well. The composite analysis, however, does not exclude possibility of intrinsic regional ocean-land-atmosphere interaction to excite the Dakar Niña.

The Dakar (Guinea) Dome is a thermal upwelling dome located near the region of interest. According to the existing literature^26^,^27^,^28^, the dome develops off Dakar seasonally from late spring to late fall owing to the Ekman upwelling^20^,^28^. Since the study period in Doi *et al.*^20^ for the Guinea Dome (1950–2001) and that for the present study (1982–2011) are different, we only consider the overlapping period 1982–2001. For this period, the cold Guinea Dome is observed in 1983, 1987 and 1991 and the warm Guinea Dome is observed in 1985. Therefore two co-occurrence years are identified; those are 1983 (cold Dakar (Guinea) Dome/Dakar Niño) and 1985 (warm Guinea (Dakar) Dome/Dakar Niña). 1987 and 1991 are years of cold Dakar (Guinea) Dome and of neutral Dakar Niño/Niña. Because of the short analysis period, however, it is difficult at this stage to derive meaningful relations between two phenomena if any.

## Discussion

The present work has investigated the interannual variability of the sea surface temperatures of the coast off Senegal from a new viewpoint of coastal Niño/Niña, which was recently introduced to explain regional climate modes confined along eastern boundaries of other basins^3^,^23^,^24^, and has shown the existence of a similar ocean-land-atmosphere coupled phenomenon for the first time in the region of interest. The phenomenon is named Dakar Niño/Niña in the present report.

For the period 1982–2011, six warm events (1983, 1984, 1997, 1998, 2008 and 2010) and five cold events (1985, 1986, 1999, 2003 and 2009) are identified. Those are locked to seasons and centred on the FMA trimester. We have suggested that regional ocean-land-atmosphere coupled dynamics may be important to explain those interesting phenomena. The important elements are the alongshore wind variability conditioning the anomalous coastal upwelling/downwelling as well as the mixed-layer depth anomaly in the upper ocean, and the modulation of the mixed-layer temperature mostly by the effective contribution from shortwave radiation. We have shown that the anomalous warming of the mixed-layer temperature during warm events is explained by warming of the anomalously thin surface mixed-layer even if the net surface heat flux contribution (mostly from solar radiation) is normal. During cold events, the reduction of the effective contribution of the net surface heat flux due to the anomalously thick surface mixed-layer and the enhanced cold water entrainment explain cooling of the mixed-layer temperature.

**Figure 8:**
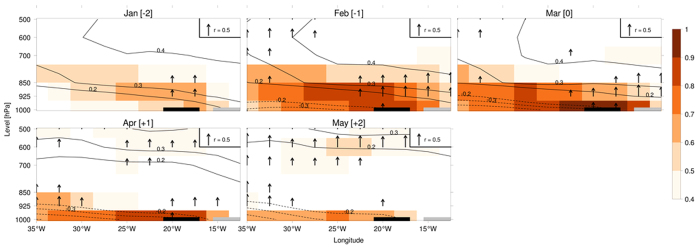
Vertical cross-section of lead-lag correlation coefficients between DNI in March and anomalies in air temperature (shading, values below 0.4 omitted), geopotential height (contour, 0 omitted) and vertical velocity (arrow). Atmospheric variables are averaged in latitude between 7.5°N and 15°N. The location of the Dakar Niño/Niña is symbolized by a black rectangle. The location of the land is symbolized by a grey rectangle. The figure was plotted by R software.

The mixed-layer temperature anomaly may influence the SST, and thus excites the cross-shore pressure gradient which is associated with the alongshore wind anomalies as observed for similar events near West Australia^24^ and California^3^. To confirm this, we have prepared Fig. 8, in which lead-lag correlation coefficients are calculated between the March DNI (symbolized with a black rectangle) and anomalies in air temperature (shading), geopotential height (contour) and vertical velocity (arrow). Atmospheric variables are averaged in latitude between 7.5°N and 15°N. During the growing and peak phases of the Dakar Niño (January to March), air temperature in the lower troposphere is anomalously heated. This warming tends to cause anomalous upward movement as well as negative SLP anomaly, enhancing the cross-shore pressure gradient. During the growing and peak phases of the Dakar Niña (January to March), air temperature in the lower troposphere is cooling, inducing downward movement as well as positive SLP anomaly, reducing the cross-shore pressure gradient. Together with the previous works^3^,^24^ on similar coastal Niño/Niña phenomena such as Ningaloo Niño/Niña and California Niño/Niña, we have suggested that the atmospheric pressure anomaly above the warm SST anomaly may generate ocean-land pressure contrast that maintains the alongshore wind anomaly and thus complete the ocean-land-atmosphere coupled feedback. The role of detailed land processes, e.g. how the humidity changes influences the cross-shore pressure gradient, is not analysed here. We have only demonstrated that the presence of the land is necessary to create the cross-shore pressure gradient so that the ocean-land-atmosphere coupled feedback may take place.

Finally, we mention that the region of the present interest is important not only for regional climate but also for regional marine ecology influencing worldwide fishery market because of active coastal upwelling. We need to have a better view of underlying physical-chemical-biological mechanisms, which eventually leads to accurate multi-variate seasonal prediction of the ocean-land-atmosphere coupled system in the region.

## Methods

The Hadley Centre Sea Ice and Sea Surface Temperature data set (HadISST) version 1.1^31^ with a 1° × 1° resolution from 1982 to 2011 is used. The choice of this period is based on sources of dataset, a blend of *in situ* observations and adjusted satellite-derived SSTs since 1982. The ocean dynamics is analysed through the use of the mixed-layer depth (MLD) from the NCEP (National Centers for Environmental Prediction) Global Ocean Data Assimilation System (GODAS) data set^32^. The mixed-layer depth is defined as the density difference between the surface and the bottom of the MLD is 0.125 kg m^−2^. GODAS dataset is also used to calculate the mixed-layer heat balance^33^. The horizontal resolution is 1° × 1°, for 40 levels in the vertical dimension. As GODAS dataset is forced by the momentum flux, heat flux and fresh water flux from the NCEP (National Centers for Environmental Prediction)/DOE (Department Of Energy) Atmospheric Model Intercomparison Project (AMIP-II) Reanalysis (NCEP2) dataset^34^, the dynamics of the atmosphere through NCEP2 reanalysis. The surface wind (10 meters) and the mean sea level pressure (SLP) are utilized to characterize the dynamics of the atmosphere near the surface. They are taken from the for the same period. The horizontal resolution is 2.5° × 2.5°. From the same dataset, air temperature, vertical velocity (changed to positive when upward) and geopotential height are used to analyse the dynamics of the atmosphere in the vertical direction between 1,000 and 500 hPa. The latent and sensible heat fluxes, as well as the longwave and shortwave radiations from NCEP2 are used to examine the air-sea interaction. In Fig. 5g, the shortwave radiation is in Wm^−2^, while in Fig. 6b–d, it is expressed as a function of the mixed-layer depth, i.e. in 10^7^ [°C s^−1^]. The sign of the latent heat flux is reversed to make its positive value downward. The total downward heat flux at surface (THF) is calculated using *Q*_*O*_*/(ρc*_*p*_*H)*, where *Q*_*O*_ is the total downward heat flux, *ρ* is the density of the seawater (function of the salinity, the temperature and the pressure), *c*_*p*_ is the specific heat of the seawater (also function of the salinity, the temperature and the pressure) and *H* is the mixed-layer depth. The THF represents the exchange of energy between the lower atmosphere and the upper ocean and is expressed in °C s^−1^. Its sign is positive when the ocean receives heat. The horizontal resolution is T62 Gaussian grid, i.e. 1.875° × 1.904°. The coastal upwelling^19^,^35^ and Ekman pumping^36^ indices are calculated from the Ekman transport derived from the NCEP2 wind stress fields. As the coastal upwelling is defined in latitudes between the equator and 20°N, it may be biased toward a slightly higher side owing to the reduced Coriolis effect^35^. The meridional surface wind at 10m is also taken from the European Reanalysis Interim (ERA-Interim) dataset^37^, with an horizontal resolution of 0.75° × 0.75°.

The NCEP2 wind product has a coarse resolution and might have some problems about the upwelling processes and the wind spatial structure near the shore^38^,^39^. However, because of the adoption of the GODAS data set, NCEP2 wind product is used for consistency.

Anomalies to the 1982–2011 period mean are calculated and the linear trend is removed from all dataset using a least-square fit. Three-month-running means are adopted for all indices to minimize the intraseasonal variations. However, similar results are found without smoothing.

The confidence level in Figs 4 and 8 is based on a random-phase test^40^. For this test, a large number of random time series (here 10,000 simulations) that have the same power spectrum as the original time series, but with random phases, are generated. The statistical significance is defined as the proportion of random correlations (coming from simulations) lower (if the observed correlation is negative) or higher (if the observed correlation is positive) than the observed correlation.

Composites in Fig. 7 are constructed for one month before and after the peak month of March. Composites in Figs 5 and 6 are constructed for five months before and after the peak month of March, i.e. from October of the previous year to August of the same year. DNI, CWI, CUI and MLDI indices are calculated in the same area as defined in Fig. 2. The components of the mixed-layer heat balance, as well as the components of the surface heat flux contribution, are also calculated in the same area. The statistical significance is based on a two-sided Student’s *t*-test, with 10,000 permutations.

## Additional Information

**How to cite this article**: Oettli, P. *et al.* A Regional Climate Mode Discovered in the North Atlantic: Dakar Niño/Niña. *Sci. Rep.* **6**, 18782; doi: 10.1038/srep18782 (2016).

## Figures and Tables

**Figure 1:**
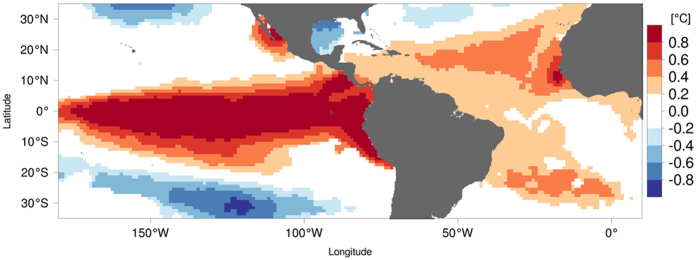
Composite of SST anomalies during January-April after the peak season of El-Niño years in the equatorial Pacific/north tropical Atlantic (period 1982–2011). The figure was plotted by R software.
